# Modified model for predicting early C-reactive protein levels after gastrointestinal surgery: A prospective cohort study

**DOI:** 10.1371/journal.pone.0239709

**Published:** 2020-09-24

**Authors:** Yui Kawasaki, Soonhee Park, Kazunori Miyamoto, Ryusuke Ueki, Nobutaka Kariya, Tsuneo Tatara, Munetaka Hirose

**Affiliations:** Department of Anesthesiology and Pain Medicine, Hyogo College of Medicine, Nishinomiya, Hyogo, Japan; University Hospital Hamburg Eppendorf, GERMANY

## Abstract

**Background:**

Postoperative serum concentration of C-reactive protein (CRP) is one of the objective quantitative indices integrating the effects of preoperative and intraoperative variables. Higher levels of CRP after gastrointestinal surgery are associated with major postoperative complications. To develop a model for predicting CRP levels on postoperative day (POD) 1 in surgical patients both with and without serious conditions and comorbidities, we modified the previous formula for prediction of CRP levels on POD1, and assessed the accuracy of our modified predictive formula for CRP levels.

**Material and methods:**

Consecutive patients of all ages undergoing gastrointestinal surgery under general anesthesia were enrolled in this single-institution prospective cohort study. We developed a modified predictive formula in a calculation cohort. Next, associations between measured CRP levels on POD1, predicted CRP levels on POD1 using the previous and modified models, and major complications after surgery were examined in a validation cohort.

**Results:**

We obtained the following model in the calculation cohort (n = 222): Modified model for predicting CRP levels on POD1 (mg•dL^-1^) = -10.13 + 0.0025 Duration of surgery (min) + 15.9 Mean Nociceptive Response (NR) + 0.66 Preoperative CRP level (mg•dL^-1^). In the validation cohort (n = 440), there was a significant association between measured and predicted CRP levels on POD1 (*P* < 0.001) No significant difference between the measured and predicted CRP levels using the modified model was observed (*P* = 0.847). There were also significant associations between the predicted CRP levels and major complications after surgery.

**Conclusion:**

CRP levels predicted using duration of surgery, mean NR, and preoperative CRP levels are likely identical to measured CRP levels on POD1, being associated with major complications after gastrointestinal surgery.

## Introduction

C-reactive protein (CRP) is an acute phase reactant produced in response to inflammation and tissue damage. Postoperative serum concentration of CRP is one of objective quantitative indices integrating the effects of preoperative comorbidities, surgical invasion, duration of surgery, anesthetic managements and analgesia [1, 2]. Early increases in serum CRP concentrations after gastrointestinal surgery reportedly associate with postoperative complications [3–10] and cancer recurrence [11, 12]. Therefore, perioperative managements aiming to prevent increases in CRP levels in the early postoperative period are relevant for better postoperative outcomes.

Higher levels of CRP on postoperative day (POD) 1 was reportedly associated with postoperative complications after gastrointestinal surgery [5, 6, 13]. Furthermore, higher levels of surgical invasion during gastrointestinal surgery increase CRP levels on POD1 [14, 15]. Previously, a model for predicting serum CRP concentrations on POD1 was developed using coefficients of the variables of duration of surgery, preoperative CRP level and Nociceptive Response (NR) as follows [16]:

$$\begin{array}{l} Previous\;model\;for\;predicting\;CRP\;levels\;on\;POD1\;(mg/dL)\\ \;=-4.38+0.0058\;Duration\;of\;surgery\;(min)+6.44\;Mean\;NR\\ \;+0.44\;Preoperative\;CRP\;level\;(mg/dL) \end{array}$$,

where mean NR value was the averaged value of NR from the start to end of surgery. NR values, which represent objective indices of autonomic responses to the balance between nociception caused by surgical invasiveness and anti-nociception due to anesthesia, are calculated using a hemodynamic equation including heart rate, systolic blood pressure, and perfusion index at arbitrary times during surgery under general anesthesia [17].

Serious preoperative conditions and comorbidities augment perioperative CRP levels and correlate with postoperative complications. The previous model for predicting CRP levels on POD1, however, was developed and validated in adult surgical patients without serious preoperative comorbidities, and its enrollment criteria were age ≥ 20 years, American Society of Anesthesiologists–physical status (ASA-PS) I or II and preoperative CRP concentration < 0.3 mg·dL-1 [16]. To improve the availability of a prediction model in surgical patients with serious conditions and comorbidities, we modified the previous model to develop a new model in a calculation cohort, and subsequently evaluated the utility of the previous and modified models in patients of all ages undergoing gastrointestinal surgery with serious conditions and comorbidities in a validation cohort in the present study. We also examined associations between major complications after surgery and CRP levels on POD1.

## Methods

This single-institutional prospective cohort study was approved by the Ethics Committee of Hyogo College of Medicine (Ethical Committee number 3138; Chairperson—Koichi Noguchi). The requirement for written informed consent for study participation was waived by the institutional ethics committee, and informed consent was obtained using an opt-out form on our institutional web-site. This study was conducted in accordance with the principles of the Declaration of Helsinki.

### Patients

This prospective cohort included all consecutive patients who underwent gastrointestinal surgery from March 2019 to February 2020 at our institutional surgical center. The exclusion criteria was patients who did not receive a routine examination of serum CRP concentration perioperatively. In a calculation cohort, consecutive patients were enrolled from March 2019 to July 2019 to modify the previous prediction formula. In a validation cohort, consecutive patients were selected from August 2019 to February 2020 to verify the value of the modified formula.

### Data collection

We collected data of serum CRP concentrations, which were measured before and after surgery on POD1 for routine perioperative examinations. The normal range for CRP at our institution is below 0.3 mg·dL^-1^. During surgery, the NR values were displayed on our institutional anesthesia information managing system every 1 min (ORSYS, PHILIPS Japan, Tokyo, Japan) [17]. The mean NR value during surgery was calculated by averaging all NR values from the start to end of surgery. Postoperative complications within 30-days after surgery were graded according to the extended Clavien-Dindo classification, which includes seven grades: grade I: any deviation from the normal postoperative course, grade II: normal course altered, grade IIIa: complications that require interventions performed under local anesthesia, grade IIIb: complications that require interventions performed under general or epidural anesthesia, grade IVa: life-threatening complications with single organ dysfunction, grade IVb: life-threatening complications with multi-organ dysfunction, and grade V: death [18]. Major complications were defined as Clavien-Dindo grade IIIa or greater.

### Surgical procedure risk

Surgical severity was divided into the three categories of low, intermediate and high procedure risk [19]. Surgical procedures with low procedure risk include appendectomy, hernia repair, laparoscopic appendectomy, laparoscopic hernia repair and laparoscopic gastric bypass. Those with intermediate procedure risk include laparoscopic colectomy, laparoscopic small bowel resection and enterostomy closure. Procedures with high procedure risk include exploratory laparotomy, repair of perforated bowel, stomach surgery, enterostomy, colectomy and small bowel resection [19].

### Anesthetic management during surgery

No patients received premedication. General anesthesia was induced with propofol, in addition to fentanyl and rocuronium, followed by insertion of a tracheal tube or supraglotic airway. Anesthesia was maintained with sevoflurane/desflurane, fentanyl, rocuronium and continuous infusion of remifentanil. Doses of remifentanil and fentanyl were adjusted to maintain mean blood pressure within the range of ± 20% of the pre-anesthesia level. Additional requirements for regional anesthesia were determined by the anesthesiologists in charge. Bispectral index was maintained between 40 and 60 by adjusting the concentration of sevoflurane/desflurane. Rocuronium bromide was used for muscle relaxation during surgery, as needed.

### Sample size calculation

The sample size in this study was calculated using software (PS Power and Sample Size Calculations, version 3.0, Dupont WD and Plummer WD). The calculation was performed based on the assumption that a type I error had a probability of 0.05 and power of 0.8. From previous studies in patients undergoing gastrointestinal surgery, which variously reported the incidence of major complications as 14.2% [20] and 28.6% [21], we assumed that the probability of major complications was 0.15. The correlation coefficient between measured and predicted CRP levels was assumed to be 0.83 (1/1.2 ≈ 0.83), and the odds ratio of major complications with CRP levels > an obtained cut-off value relative to those ≤the cut-off value was estimated to be 3, based on the previous study on predicting CRP levels on POD1 [16]. Finally, the sample size was estimated to be 435 patients in the present study. Thereafter, we enrolled 440 patients in the validation cohort.

### Statistics

Comparisons of two variables were performed using the chi-square test or one-way ANOVA followed by the Tukey’s post-hoc test for appropriate variables. Linear regression analysis was performed to evaluate relationships between measured and predicted CRP levels. Agreement between measured and predicted CRP levels was assessed by the Bland-Altman analysis. Receiver-operating characteristic (ROC) curve analysis was used to evaluate associations between major complications and each CRP level. The statistically significant level was considered as *P*<0.05. All statistical analyses were performed using JMS Pro version 14.2.0 (SAS Institute Inc. Cary, NC, United States). All values were reported as mean ± SD.

## Results

The characteristics of all enrolled patients in the calculation cohort (n = 222) and validation cohort (n = 440) are shown in Table 1. There were no significant differences in perioperative variables between the two cohorts. No significant difference was also observed in the incidence of major complications, defined as Clavien-Dindo grade IIIa or greater, between 14.0% (31/222) in the calculation cohort and 16.6% (73/440) in the validation cohort (*P* = 0.387).

**Table 1:** Patient demographics and perioperative variables.

Perioperative variables		Calculation cohort	Validation cohort	*P*
		n = 222	n = 440	
Preoperative variables	Age, yrs	60 ± 19	58 ± 20	0.180
	Male / female, n (%)	127 / 95 (57 / 43)	278 / 162 (63 / 37)	0.136
	Body mass index, kg·m^-2^	21.2 ± 3.7	21.4 ± 4.1	0.635
	ASA-PS, I / II / III / IV / V, n (%)	19 / 143 / 58 / 2 / 0 (9 / 64 / 26 / 1 / 0)	28 / 289 / 116 / 7 / 0 (6 / 66 / 26 / 2 / 0)	0.669
	Elective / emergency surgery, n (%)	189 / 33 (85 / 15)	373 / 67 (85 / 15)	0.902
	Surgical procedure risk: Low / Intermediate / High, n (%)	60 / 99 / 63 (27 / 45 / 28)	88 / 200 / 152 (20 / 45 / 35)	0.082
	Preoperative CRP level, mg·dL^-1^	2.21 ± 6.06	2.10 ± 5.48	0.808
Intraoperative variables	Duration of surgery, min	209 ± 138	207 ± 162	0.874
	Blood loss, mL	172 ± 370	162 ± 328	0.785
	Mean NR	0.812 ± 0.054	0.817 ± 0.060	0.349
Postoperative variables	CRP level on POD1, mg•dL^-1^	4.81 ± 5.65	4.82 ± 5.52	0.973
	Clavien-Dindo class, No complications / I / II / IIIa / IIIb / IVa / IVb / V, n (%)	69 / 73 / 48 / 19 / 5 / 4 / 0 / 3 (31 / 33 / 22 / 9 / 2 / 2 / 0 / 1)	95 / 178 / 95 / 41 / 11 / 6 / 5 / 8 (22 / 41 / 22 / 9 / 2 / 1 / 1 / 2)	0.155

ASA-PS: American Society of Anesthesiologists–Physical Status, BMI: body mass index, CRP: C-reactive protein, NR; Nociceptive Response, POD: postoperative day.

To modify the previous model for predicting CRP levels on POD1 in the calculation cohort, we performed linear regression analysis to model the relationship between the measures CRP levels on POD1 as a dependent variable and three variables of duration of surgery, mean NR value and preoperative CRP level as independent variables, which were the same variables used in the previous model [16]. Thereafter, we developed a new model as follows:

$$\begin{array}{l} Modified\;model\;for\;predicting\;CRP\;levels\;on\;POD1\;(mg/dL)=\\ \;=-10.13+0.0025\;Duration\;of\;surgery\;(min)\\ \;+15.9\;Mean\;Nociceptive\;Response\;(NR)\\ \;+0.66\;Preoperative\;CRP\;level(mg•dL-1). \end{array}$$

**Fig 1:**
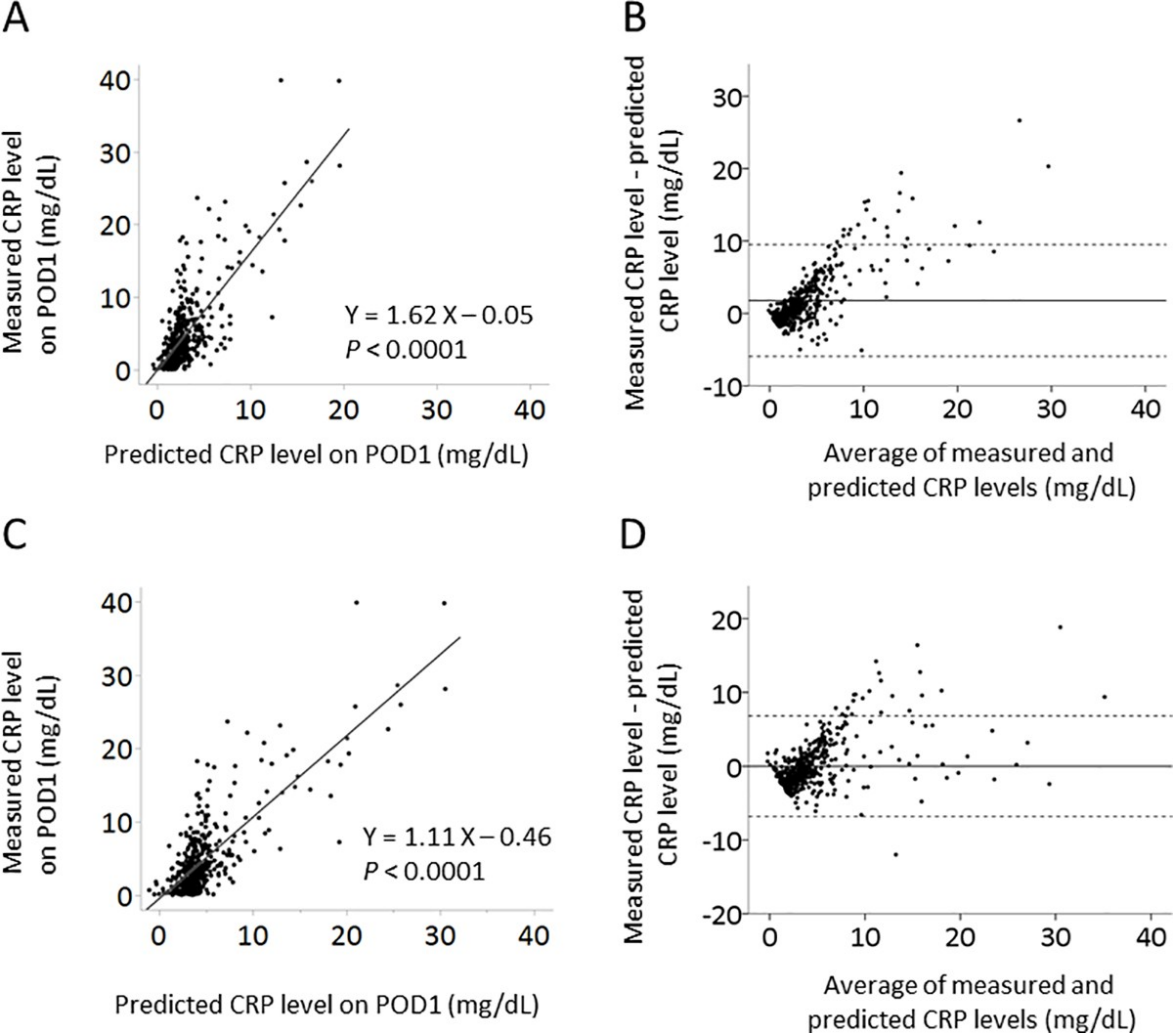
Relationships between predicted and measured CRP levels on POD1. Scatter-points represent actual data of measured and predicted CRP values using the previous model (A) and modified model (C). In the Bland-Altman plot of measured and predicted CRP levels on POD1 using the previous model (B) and modified model (D), continuous line shows mean difference in measured and predicted CRP levels on POD1, while dotted line shows upper and lower limits of agreement. CRP: C-reactive protein, POD: postoperative day.

Next, we compared measured and predicted CRP levels in the validation cohort. There were significant associations between measured and predicted CRP levels using the previous model (*P* < 0.001, Fig 1A), and between measured and predicted CRP levels using the modified model (*P* < 0.001, Fig 1C). Although the predicted CRP levels of 3.01 ± 2.62 mg·dL^-1^ using the previous model were significantly lower than the measured CRP levels of 4.82 ± 5.52 mg·dL^-1^ (*P* < 0.001), the predicted CRP levels of 4.76 ± 3.93 mg·dL^-1^ using the modified model were not significantly different from the measured CRP levels (*P* = 0.847). Bland-Altman plot also confirmed the better clinical validity of the modified model than the previous model, showing a good agreement between measured and predicted CRP levels using the modified model with fixed bias between measured and predicted CRP levels (*P* < 0.0001 in the previous model, Fig 1B, and *P* = 0.700 in the modified model, Fig 1D).

**Fig 2:**
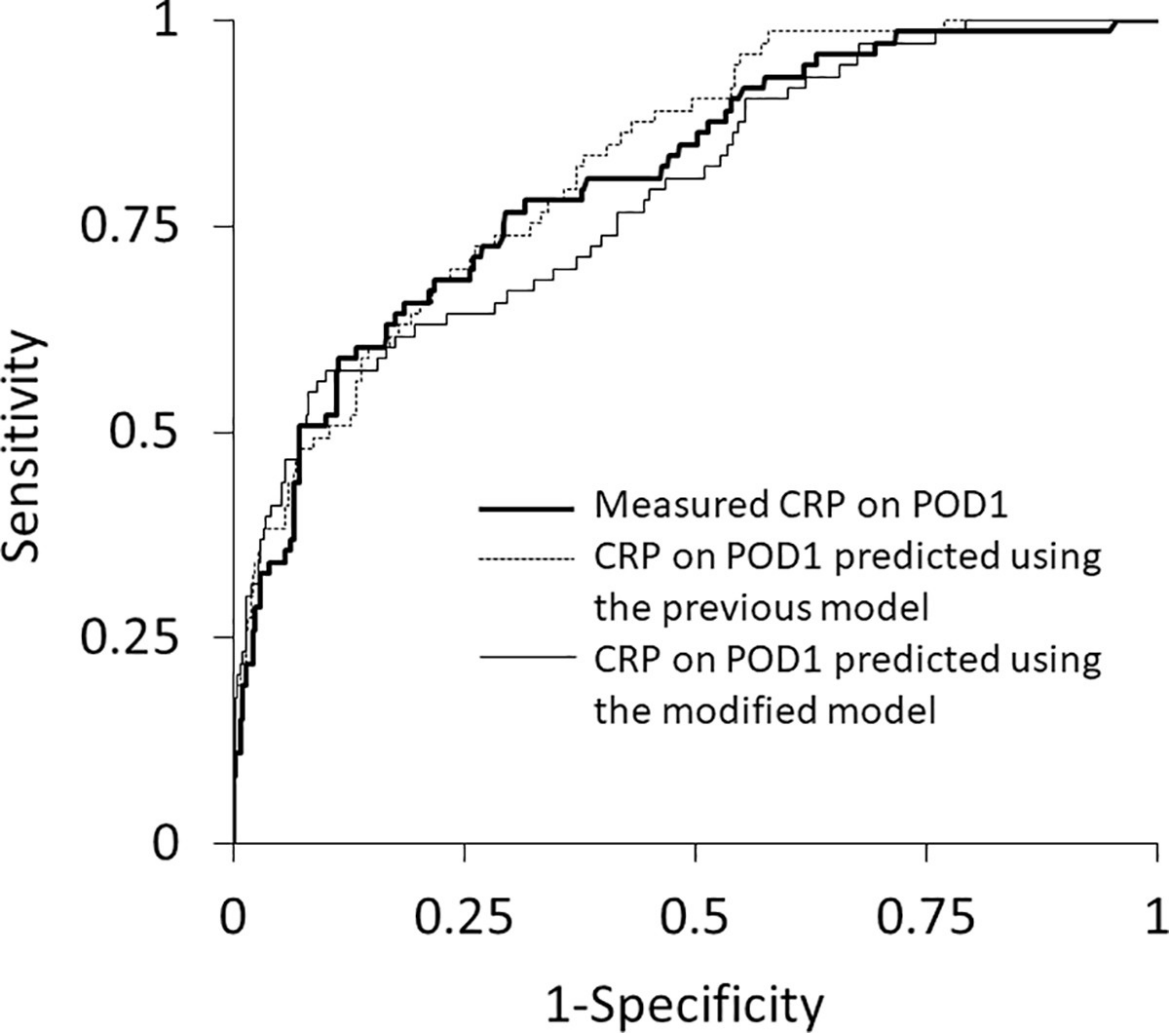
Receiver operating characteristic curve analysis between predicted and measured CRP levels on POD1 and postoperative major complications. Receiver operating characteristic curves for major complications (Clavien-Dindo grade IIIa or greater) versus measured CRP on POD1 (bold line), and predicted CRP on POD1 using the previous model (dotted line) and modified model (black line). CRP: C-reactive protein, POD: postoperative day.

ROC curve analysis revealed that all three values of measured CRP and predicted CRP using previous and modified models showed significant associations with major complications after gastrointestinal surgery (Table 2, Fig 2). Calibrations also showed significant associations between major complications and each CRP value > the cut-off values, which were 7.24 mg·dL^-1^ in the measured CRP level and 5.40 mg·dL^-1^ in the predicted CRP level using the modified model. There were no significant differences in the area under the curve (AUC) values between these three CRP values (Table 2).

**Table 2:** Receiver operating characteristic curve analysis between CRP levels and major complications (Clavien-Dindo class ≥ IIIa) in the validation cohort.

CRP	AUC [95% CI]	Sensitivity	Specificity	Cut-off value	Odds ratio for calibration [95% CI]
Measured CRP on POD1 (mg•dL^-1^)	0.808 [0.746–0.857]	0.589	0.885	7.24	11.06 [6.28–19.48] ^‡‡^
CRP on POD1 predicted using the previous model (mg•dL^-1^)	0.824 [0.769–0.868]	0.685	0.865	3.02	7.77 [4.47–13.50] ^‡‡^
CRP on POD1 predicted using the modified model (mg•dL^-1^)	0.790 [0.725–0.843]	0.575	0.899	5.40	11.39 [6.42–20.22] ^‡‡^

AUC: area under the curve, CI: confidence interval, CRP: C-reactive protein, POD: postoperative day. There were no significant differences in AUC values between three CRP values.

^‡‡^*P*<0.001, significant in the Chi-square test for calibration.

## Discussion

CRP levels on POD1 predicted using the previous and modified formulas were significantly associated with measured CRP levels and major complications after gastrointestinal surgery in the present study. The variables in predictive CRP formulas include duration of surgery, preoperative CRP levels and mean NR. The NR value represents autonomic responses to the balance between nociception caused by surgical invasion and anti-nociception provided by general anesthesia [17]. Given that higher values of mean NR, which were calculated by averaging NR values from the start to end of surgery, are reportedly associated with an increased incidence of postoperative complications in laparoscopic gastrointestinal surgery [22], it seems plausible that mean NR is included in a prediction formula for calculating postoperative CRP levels.

Since predicted levels of CRP on POD1 using the previous model shifted significantly lower than measured CRP levels in patients undergoing gastrointestinal surgery, the previous model was corrected to the modified model in the present study. Analysis showed that predicted CRP levels with the modified model showed better associations with measured CRP levels than did predicted CRP levels with the previous model. The value of the coefficient for duration of surgery in the linear prediction formula decreased from 0.0058 in the previous model to 0.0025 in the modified model. On the other hand, the coefficient values for mean NR and preoperative CRP level in the linear formula increased from 6.44 and 0.44, respectively, in the previous model to 15.9 and 0.66, respectively, in the modified model. Serum CRP levels are reportedly affected by the patient’s preoperative condition, tissue injury, surgical procedure, duration of surgery and perioperative management [1, 2, 16]. Therefore, serious preoperative conditions and comorbidities would augment mean NR and preoperative CRP values. Since the previous model was developed and validated in patients without serious preoperative conditions and comorbidities [16], predicted CRP levels using the previous model in all patients, including those with serious preoperative conditions and comorbidities, might be lower than measured CRP levels, as was seen in the present study.

The prediction formula for CRP levels on POD1 included three coefficient variables of duration of surgery, mean NR and preoperative CRP levels. Therefore, shortening of duration of surgery, perioperative management to suppress CRP levels, and intraoperative management to keep the NR values as low as possible during surgery might be effective for prevention of major complications after surgery. Although anesthetic agents or techniques, including regional anesthesia, volatile anesthetics or intravenous anesthetics, reportedly showed no effects on CRP levels after surgery [23–26], the Fast-Track Surgery (FTS) and the Enhanced Recovery After Surgery (ERAS) suppressed CRP levels on POD1 in the previous reports [27, 28]. Intravenous injections of esmolol [29, 30], steroid [31, 32] and flurbiprofen [33] during and after surgery were reported to suppress postoperative CRP levels. Therefore, predicted CRP levels on POD1 using the modified model ≥ 5.40 mg·dL^-1^ might be an index to consider administering these agents during or early after surgery for prevention of postoperative complications even a little.

It might be possible that a large deviation between measured and predicted CRP levels using the modified model occurs. At this time, an unexpected adverse event could be a cause of this deviation. Further investigations are needed to investigate causality and mechanism of large deviations between measured and predicted CRP levels.

A limitation of this study is that the present study was performed in patients undergoing gastrointestinal surgery, where the incidence of major complications was 14.0% in our calculation cohort and 16.6% in our validation cohort. On the other hand, the reported incidences of major complications were 3.9% after mastectomy [34], 8.8% after non-laparoscopic gynecological surgery [35], 13.1% after radical prostatectomy [36], and 28.6% after major abdominal surgery [21]. Further study is needed to confirm the versatility of the modified model for predicting CRP levels on POD1 following different types of surgeries, since the influence of the variables of duration of surgery, mean NR and preoperative CRP levels on predicted CRP levels might differ depending on the surgical procedure performed.

## Conclusion

CRP levels predicted using the modified model are likely identical to measured CRP levels on POD1 after gastrointestinal surgery in patients both with and without serious conditions and comorbidities.

## Supporting information

S1 File (XLSX)
